# Comparison of Ponseti and Kite's method of treatment for idiopathic clubfoot

**DOI:** 10.4103/0019-5413.61941

**Published:** 2010

**Authors:** Raju Rijal, Bikram Prasad Shrestha, Girish Kumar Singh, Mahipal Singh, Pravin Nepal, Guru Prasad Khanal, Pramila Rai

**Affiliations:** Department of Orthopedics, BPKIHS, Dharan, Nepal; 1 CSSM, Lucknow, India; 2 Public Health and Community Medicine, BPKIHS, Dharan, Nepal

**Keywords:** Clubfoot, kite, Ponseti, manipulation, Pirani score

## Abstract

**Background:**

The manipulation and corrective cast application for club foot was known to be done by Kite's method. The Kite's method of manipulation (center of rotation of malaligned foot and fulcrum on cuboid) was modified by Ponseti (fulcrum on head of talus). Recently, Ponseti's method has gained popularity and vastly improved results are reported. We report randomized controlled trial where manipulation of club foot was done by Ponseti's and Kite's method and correction evaluated by Pirani score to compare the outcome of treatment.

**Materials and Methods:**

Sixty feet in 38 patients, 22 with bilateral and 16 with unilateral clubfeet in children less than two years of age and without any prior manipulation or surgical treatment were randomly allocated to the Ponseti (30 feet) and Kite (30 feet) methods of manipulation. This process resulted in the right and left feet of the same patient in 12 bilateral cases being compared with one another (Paired analysis). In the remaining 10 bilateral cases, four patients had both feet treated by Ponseti and six had both feet treated by Kite (unpaired analysis). Finally, in 16 unilateral cases, 10 feet were allocated to the Ponseti and six to Kite methods of manipulation (unpaired analysis). Feet were followed up weekly for 10 weeks for change of cast and recording of hindfoot, midfoot and total Pirani scores. Correction was measured as a difference between hindfoot, mid foot and total Pirani scores weekly from weeks 1 to 10 and corresponding baseline scores. Absolute correction and rate of correction in (i) bilateral clubfeet treated by Ponseti's method on one side and Kite's method on the other side in the same patient were compared using paired Student's t test and (ii) patients with unilateral clubfoot (where either of the methods was used) or those with bilateral clubfoot (where both feet treated by either of the two methods on both the sides) were compared using difference between means (mean correction by Ponseti minus mean correction by Kite) for magnitude of difference and unpaired Student's t test (if data was normally distributed) or Mann Whitney U statistics (otherwise) for significance of difference.

**Results:**

In 12 bilateral clubfeet, where one foot received Kite's method and the other Ponseti's manipulation, feet treated by Ponseti's technique showed faster rates of decrease in Pirani score (improvement) as compared to feet treated by Kite's method with the mean of difference between baseline and follow up scores showing significantly greater (*P*<0.05) difference from zero from fourth week onwards to up to 10 weeks. In unpaired analysis, both for unilateral or bilateral clubfeet, regardless of side, mean Pirani scores in Ponseti feet improved much faster than Kite feet but the difference achieved statistical significance only at the 10^th^ week from the start of treatment.

**Conclusions:**

Hind foot, midfoot and total Pirani scores reduce much faster with Ponseti than the Kite's method of manipulation of clubfoot. In paired analysis the difference becomes statistically significant at fourth week and in unpaired analysis at 10^th^ week from the start of treatment.

## INTRODUCTION

The treatment of idiopathic congenital clubfoot is serial gentle manipulations to stretch the contractures, with serial casting, splinting, or strapping to maintain the correction.^1^ The goal of treatment is to achieve a functional, pain-free, plantigrade foot with good mobility and without calluses.^2^ Non-operative serial manipulation and casting, as described by Kite (1939), was used for a long time in the past.^1^,^3^ The reported success rates were only fair, ranging from 11 to 58%.^1^ Ponseti method (1950) of serial manipulation and casting has recently been used.^4^,^5^ Ponseti claims to avoid open surgery in 89% of cases by using his technique of manipulation, casting, and limited surgery.^6^ Cooper and Dietz reviewed Ponseti's cases, with an average of 30 years of follow-up, and found that 78% of the patients had achieved excellent or good functional and clinical outcomes compared with 85% in a control group without congenital foot deformity.^7^

Of the two methods of manipulation, i.e. Ponseti and Kite, which method can give a lasting and better outcome is a dilemma. The only one randomized controlled trial (RCT) available in literature consisting of 45 infants (67 feet) younger than three months^8^. The study concludes that Ponseti's method is superior to Kite's method in achieving correction in idiopathic clubfeet in a relatively shorter period of time when used to treat young infants. However, with both Kite and Ponseti methods being manipulation techniques, correction in scores and its relation to time to achieve correction has not been quantified. Hence we decided to conduct an RCT to compare the outcome of idiopathic clubfoot by Ponseti and Kite method of manipulation.

## MATERIALS AND METHODS

This prospective randomized controlled trial, conducted after obtaining approval from the Institutional ethics and research committee, compared Kite and Ponseti methods of manipulation and casting in idiopathic congenital clubfoot. Out of 49 clubfeet patients attending our outpatient department between July 2005 and May 2006, 38 were included in the study [22 bilateral and 16 unilateral (60 feet)]. Cases with age more than 2 years (n=6) and cases having received prior surgically intervention (n=5) were excluded.

Thus 60 feet were randomized to Ponseti or Kite methods of manipulation (30 in each group) using computerized random number generation technique on Microsoft Office Excel 2007. The randomization process with foot as a unit resulted in three subpopulations of feet needing different type of analysis strategies. Twelve patients with bilateral clubfeet, who had received Ponseti's method of manipulation on one side and Kite's method on the other, were subjected to paired analysis. Four of the 10 patients with bilateral clubfeet who received Ponseti method in both feet and the other six who received Kite method in both feet were subjected to unpaired analysis. Ten of the sixteen cases with unilateral clubfeet who received Ponseti method and the other six who received the Kite's method of treatment were subjected to unpaired analysis.

Patients were examined as per standardized pilot tested proforma and severity of clubfoot was noted according to Pirani clubfoot score.^9^ Kite's method^3^,^10^ and Ponseti's^11^ method of manipulation and casting were performed as per randomization method. The margin of the cast was checked to avoid any skin impingement. Post plaster neurovascular assessment was done. Parents were cautioned to look for the complications of cast like swelling, bluish discoloration of the toes and excessive cry of the baby.

Follow-up was done at weekly intervals for 10 weeks. At each follow-up, foot was evaluated for deformity correction using Pirani score for hinfoot, midfoot and total scores, any associated pressure sore or swelling of limb. In both method Tendo Achilles tenotomy was done if the equinus as per the treating surgeons judgement was not passively correctible without danger of producing a Rocker Bottom foot or Table Top Talus to treat the remaining equinus deformity. In both methods, after the last cast was removed, all children were treated with abduction splint with shoes having straight and stiff medial border to correct forefoot adduction. Till child could not walk, the two shoes were connected by a Bar (Dennis Brown) set at 45° external rotation for a normal foot and 70° for the clubfoot. For bilateral cases, both feet were set at 70° of external rotation. Abduction orthosis, was applied for 23 hours per day, for the first three months and then at night time only for two to four years. Once the child started walking, custom made clubfoot shoes were used. Patients not having satisfactory correction at the end of 10th week were subjected to operative methods of deformity correction. Difference between Pirani score at start of treatment (baseline score) at weekly follow-up was recorded and the measurement was compared between groups. Pirani score was calculated separately for the hind and the mid foot with a higher score means greater deformity. Change of hind foot, midfoot and total Pirani scores at each weekly follow-up from one to 10 weeks was calculated by subtracting the score at follow up from the initial baseline score and the value was recorded. A greater negative value signified greater correction. Bonferroni correction was used to know the significance level.

Twelve patients with bilateral clubfeet, who had received Ponseti method on one side and Kite's method on the other side, were subjected to paired analysis where hind foot, midfoot and total pirani scores at each week (till 10 weeks) were compared between the two sides. In 10 bilateral clubfeet, both feet of the same patient were randomized to Ponseti (n=4) or Kite's method of treatment (n=6) were subjected to unpaired analysis. In 16 cases with unilateral clubfoot which received either Ponseti (n=10) or Kite's method (n=6) were subjected to unpaired analysis.

Mean of the foot wise differences from baseline at follow-up week 1 to 10 (Correction 1–10) between Ponseti and Kite treated should have been 0 according to the null hypothesis. If Ponseti correction minus Kite Correction was positive and significantly different from zero (*P* value by non parametric Mann Whitney U statistics) and negative (below zero) then it was interpreted that Ponseti method was doing better and the result could be generalized to target population statistically significant

For bilateral clubfeet where both feet treated were either by Ponseti or Kite's method or for unilateral clubfeet on right or left side, the values of means of improvement at various follow-ups were compared amongst feet treated by Ponseti or Kite's method to determine the magnitude of difference (difference between means) and significance of difference by *P* value calculated vide Mann Whitney U statistics.

## RESULTS

### Paired analysis

Feet being compared belong to the same patient (n=12 patients one foot treated by Ponseti method and other foot by Kite method).

### Hind foot scores in 12 patients with Bilateral clubfeet

Table 1 shows that the Ponseti scores are decreasing faster than Kite and the final scores at 10 weeks by Ponseti method (0.7) showed less deformity than with Kite's method (1.31). (The correction [mean initial score minus the score at follow-up] when compared at each week of follow-up shows, larger negative values thereby meaning that Ponseti's method was able to achieve better correction that increased successively till the end of week 10 and the difference gained significance at week 4 (*P*=0.006). Bonferroni correction was used to know the level of significance which was at *P*<0.005.

**Table 1:** Pirani club foot score of bilateral clubfoot one foot treated by Ponseti and other by Kite method 12 patients- 24 feet

Weeks	Hindfoot mean±SD			Midfoot mean±SD			Total mean±SD		
			
	Ponseti	Kite	*P* value*	Ponseti	Kite	P*	Ponseti	Kite	P*
1	2.62±0.48	2.79±0.33	0	2.62±0.48	2.7±0.33	0	05.2±0.96	5.5±0.64	0.05
2	2.37±0.52	2.58±0.46	0.16	2.29±0.5	2.41±0.28	0.16	4.58±1.01	5±0.70	0.07
3	2±0.52	2.25±0.45	0.16	2±0.52	2.25±0.39	0.01	4.04±1.05	4.29±0.92	0.03
4	1.7±0.58	2.16±0.49	0.006	1.67±0.53	2.12±0.48	0.002	3.37±1.08	4.29±0.92	0.02
5	1.59±0.43	2.04±0.49	0.005	1.4±0.49	1.87±0.43	0.001	3±0.86	3.91±0.87	0.001
6	1.36±0.45	1.83±0.44	0.003	1.13±0.45	1.75±0.54	0.001	2.5±0.86	3.58±0.95	0.001
7	1.04±0.47	1.7±0.45	0.002	0.95±0.41	1.54±0.45	0.001	2±0.80	3.25±0.84	0.001
8	1±0.14	1.63±0.16	0.002	0.82±0.37	1.5±0.38	0.0001	1.81±0.70	3.13±0.71	0.0001
9	0.7±0.27	1.5±0.32	0.0001	0.8±0.27	1.13±0.39	0.0004	1.5±0.5	2.63±0.67	0.0001
10	0.7±0.27	1.31±0.40	0.0001	0.5±0.35	1.04±0.35	0.0001	1.2±0.57	2.36±0.67	0.0001

* Bonferroni correction-*P*<0.005 is considered as a significance

### Mid foot scores in 12 patients with bilateral clubfeet

Table 1 shows that the Ponseti scores decrease faster than the Kite and the final scores at 10 weeks by Ponseti (0.5) show less deformity than with Kite (1.04). The correction (initial score minus the score at follow-up) when compared at each week of follow-up, yielded larger negative values thereby meaning that Ponseti's method was doing better and improvement in score started from 0 at 1^st^ week to-0.75 at 10^th^ week and the difference gained significance at 4^th^ week (*P*=0.0001).

### Total scores in 12 patients with bilateral clubfeet

Table 1 shows that the Ponseti scores are decreasing faster than Kite and the final scores at 10 weeks by Ponseti (1.2) shows less deformity than with Kite (2.36). The correction, (initial score minus the score at follow-up) when compared at each week of follow-up, yielded larger negative values thereby meaning that Ponseti was doing better and improvement in score started from 0.05 at 1^st^ week to-1.54 at 10^th^ week and the difference gained significance at 4^th^ week *P*=0.0001.

### Unpaired analysis: Feet being compared belonged to different patients

In all subgroup comparisons, when right sides were compared to right and left in bilateral clubfeet [Table 2] and in unilateral clubfeet, regardless of side [Table 3], similar trend as above was witnessed.

**Table 2:** Mean Pirani score, standard deviation and *P* value of bilateral clubfeet both side treated by same method; either Ponseti or Kite method

	Hindfoot			Midfoot			Total			Hindfoot			Midfoot			Total		
						
Weeks	Ponseti	Kite	P*	Ponseti	Kite	P*	Ponseti	Kite	P*	Ponseti	Kite	P*	Ponseti	Kite	P*	Ponseti	Kite	P*
Initial	2.88±0.25	2.58±0.66	0.6	2.75±0.28	2.67±0.52	1	5.63±0.48	5.25±1.17	1	2.88±0.25	2.58±0.67	0.6	2.87±0.25	2.67±0.52	0.59	5.75±0.29	5.25±1.17	1
1	2.87±0.25	2.58±0.66	0.6	2.75±0.28	2.67±0.52	1	5.63±0.48	5.25±1.17	1	2.88±0.25	2.58±0.67	0.6	2.87±0.25	2.67±0.52	0.59	5.75±0.29	5.25±1.17	1
2	2.50±0.40	2.41±0.58	0.9	2.63±0.48	2.25±0.18	0.2	5.13±0.85	4.67±0.98	0.44	2.5±0.41	2.5±0.63	0.82	2.5±2.	2.33±0.52	0.59	5±0.41	4.83±1.1	0.82
3	2.25±0.28	2.16±0.40	0.8	2.38±0.75	2.17±0.52	0.5	4.62±1.03	4.33±0.88	0.44	2.13±0.25	2.33±0.52	0.36	2.125±0.25	2.25±0.42	0.41	4.25±0.29	4.58±0.92	0.27
4	2.21±0.47	1.91±0.37	0.4	1.88±0.48	2±0.45	0.65	4±0.91	3.91±0.80	0.82	1.88±0.25	2.08±0.58	0.25	1.63±0.25	2±0.63	0.26	3.5±0.41	4.08±1.2	0.23
5	1.75±0.64	1.67±0.25	0.8	1.75±0.65	1.58±0.38	0.65	3.62±1.11	3.5±0.71	0.91	1.88±0.25	1.75±0.69	0.1	1.38±0.25	1.83±0.52	0.12	3.25±0.5	3.58±1.15	0.28
6	1.62±0.75	1.58±0.37	0.7	1.5±0.41	1.41±0.38	0.73	3.13±1.11	3.08±0.58	0.91	1.25±0.65	1.66±0.61	0.25	1.38±0.25	1.58±0.49	0.43	2.62±0.75	3.25±1.15	0.23
7	1.37±0.85	1.58±0.37	0.5	1.38±0.63	1.25±0.27	0.49	2.75±1.3	2.83±0.61	0.67	1±0.41	1.58±0.58	0.09	0.88±0.48	1.33±0.26	0.1	1.87±0.75	2.91±0.80	0.07
8	1.12±0.75	1.25±0.42	0.7	0.88±0.48	1.08±0.38	0.43	2±1.22	2.33±0.75	0.58	0.88±0.48	1.42±0.49	0.1	0.87±0.21	1.25±0.42	0.81	1.5±0.10	2.66±0.88	0.12
9	0.87±0.47	1.17±0.25	0.2	0.63±0.25	1±0.32	0.08	1.5±0.58	2.17±0.52	0.1	0.63±0.25	1.33±0.41	0.02	0.5±0.41	1.08±0.38	0.05	1.12±0.63	2.41±0.73	0.02
10	0.63±0.25	1.08±0.2	0.02	0.38±0.25	0.92±0.20	0.01	1±0.41	2±0.32	0.01	0.38±0.25	1.16±0.41	0.01	0.08±0.25	1.0±0.32	0.01	0.62±0.48	2.1±0.68	0.01

* Mann Whitney U statistics *P* value <.05 =significance, Ponseti- 4 patients= 8feet, Kite- 6 patients= 12 feet

**Table 3:** Total mean Pirani score, standard deviation and Mann Whitney *P* value of unilateral clubfoot treated by Ponseti or Kite method (16 patients-16 feet)

Weeks	Hindfoot mean±SD			Midfoot mean±SD			Total mean±SD		
			
	Ponseti**	Kite***	P*	Ponseti**	Kite***	P*	Ponseti**	Kite***	P*
Initial	2.85±0.33	2.58±0.58	0.26	2.90±0.31	2.67±0.60	0.23	5.75±0.63	5.25±1.17	0.2
1	2.75±0.42	2.58±0.58	0.49	2.85±0.33	2.67±0.60	0.52	5.6±0.73	5.25±1.17	0.45
2	2.45±0.59	2.33±0.51	0.57	2.50±0.47	2.33±0.51	0.48	4.95±1.01	4.66±0.93	0.4
3	2.2±0.48	2.17±0.68	0.95	2.15±0.47	1.92±0.58	0.49	4.35±0.88	4.08±1.24	0.74
4	1.85±0.62	1.83±0.81	0.86	1.80±0.48	1.83±0.60	0.9	3.65±1.08	3.6±.1.32	0.91
5	1.70±0.67	1.67±0.68	0.86	1.50±0.57	1.58±0.38	0.73	3.2±1.22	3.25±1.29	1
6	1.30±0.71	1.42±0.58	0.69	1.28±0.53	1.58±0.66	0.21	2.5±1.17	3±1.22	0.47
7	1.10±0.61	1.33±0.60	0.43	0.85±0.57	1.33±0.51	0.11	1.95±1.14	2.6±1.03	0.19
8	1.0±0.47	1.25±0.52	0.75±0.48	1.25±0.41	0.3	0.04	1.75±0.92	2.5±0.83	0.06
9	0.70±0.42	1.08±0.37	0.50±0.25	1±0.54	0.08	0.03	1.15±0.57	2.08±0.86	0.03
10	0.60±0.31	0.91±0.20	0.04	0.45±0.28	1±0.54	0.02	1.05±0.49	1.91±0.73	0.02

* Mann Whitney U statistics *P* value

** 10 feet

*** 6 feet

Though the mean hind foot deformity was more with Ponseti's technique, it became less at six weeks; statistical significance was however achieved only at the 10^th^ week (*P*=0.02).

Right sided midfoot Pirani score and total Pirani score analysis showed that though the mean Pirani score from 1–7 weeks of follow-up in the Ponseti and the Kite group feet were more or less similar, 8 weeks onward the deformity by Ponseti's method showed faster improvement and gained statistical significance (*P*=0.01) at the tenth week.

## DISCUSSION

Congenital Talipes Equino Varus (CTEV) deformity is a relatively common congenital anomaly, the incidence ranging from 1–2 per thousand births. Boys are affected twice as often as girls and the condition is bilateral in one-third of cases.^12^ In our study, 57.89% were bilateral, 76.2% were male and among unilateral involvement 68.75% were right. One patient was associated with developmental dysplasia of hip. Most children (90%) were full term without complications at gestation and delivery. None of our study patients had positive family history of clubfoot as contrast to other studies some of which shows up to 22% of positive family history of clubfoot.^13^

Mean age at treatment was 195.7±202.81 days (3–720 days). Our protocol was to treat the patients as soon as they attended the OPD, as the deformed small bones (mainly the talar head and neck) are composed of young cartilage and can be remodeled easily when treatment is started early.^2^ Such rapid remodeling has been shown by Magnetic Resonance Imaging image of clubfeet that were treated by Ponseti method at ages 2 months, 2.5 months, and 3 months.^14^

In our study, the trends of Pirani score in hind foot, midfoot and total score showed decrease (improvement of deformity) from the initial period at weekly follow-up to the last follow-up of 10^th^ weeks in both methods but the rate of decrease was much faster with Ponseti as compared to the Kite method. In cases of bilateral clubfeet where one foot was treated by Ponseti method and the other by Kite method, mean hind foot Pirani score at initial period before casting was 2.62 in Ponseti method and 2.79 in limbs treated by kite's method. This decreased to 0.7 and 1.31 respectively at the 10^th^ follow-up. The mean of difference between two group was statistically highly significant (*P*<0.0001) 4^th^ weeks onwards. Similarly in this group of patients midfoot score by Ponseti method and Kite method of treatment reduced from initial 2.62 and 2.7 to 0.5 and 1.04 respectively mean of difference different from with *P*<0.05. In total score of 5.2 and 5.5 by Ponseti and Kite treatment method reduced to 1.2 and 2.36 respectively at 10^th^ follow-up (*P*=0.0001).

In cases of bilateral clubfeet in which both feet were treated either with Ponseti or Kite method was assessed weekly with Pirani score for hind foot, midfoot and total score for right and left limb were analyzed separately. Analysis shows that no significant difference at distribution of age and sex between the groups. Right sided analysis shows total Pirani score of 5.63±0.48 in the Ponseti group and 5.25±1.17 in the Kite group that reduced to 1±0.41 and 2±0.32 respectively, with *P* value 0.01 at tenth week of follow up. In left sided analysis Ponseti and Kite group total score was reduced from 5.79±0.29 and 5.25±1.17 to 0.62±0.48 and 2.1±0.68 respectively *P* value 0.01.

In case of unilateral clubfoot treated by Ponseti method total Pirani score of 5.75±0.63 reduced to 1.05±0.49 at tenth follow up. 5.25±1.17 of score in patients treated by Kite reduced to 1.91±0.73, *P* value 0.02.

As decrease in Pirani score means improvement and reduction in the components of clubfoot deformities, our study indicates that Ponseti method of manipulation and casting was superior in reduction and correction of clubfoot deformity as compared to the Kite method of manipulation and casting. Our results are comparable with other studies in the literature in terms of Ponseti method being better than the Kites method.^1^,^5^,^7^,^13^–^21^ but none of them have directly compared the two methods.

Among the Ponseti group, 29 out of 30 (96%) underwent percutaneous tendo Achilles tenotomy before correction of equines deformity, There were no post tenotomy complications like excessive bleeding, swelling, infection, skin necrosis. Our results are comparable with study conducted by Morcuende *et al*, in which 86% required percutaneous tendo Achilles tenotomy.^13^

This study is unique in the sense that it quantifies rates of correction in hind foot and midfoot deformities, over a 10-week period, measured by Pirani score, in cases of clubfeet. It compares the rates and quantifiably proves that the rate of reduction of hind foot and midfoot deformity is higher with Ponseti method as compared to Kite method and the difference reaches statistical significance at four weeks in paired study and 10 weeks in unpaired study.

Random allocation was successful as shown by similar distribution of possible confounders like age, sex and baseline Pirani scores in the two groups of feet receiving Ponseti or Kites manipulation. This annuls the possibility of these or other known or unknown confounders explaining the difference. Since measurements were by objective scores on podograms by blinded observers’ measurement bias has been avoided. Since all consecutive cases in one year have been included and since there is no referral filter at our institution the cases representing the source and the target population is a reasonable assumption decreasing the possibility of a selection bias. The sample size is adequate as even in subgroups the differences have attained statistical significance. The follow-up is adequate because of the same reason. Contamination, co intervention, lack of compliance was simply not possible. Thus the observed results are solely attributable to difference in the manipulation technique, are internally valid, have sufficient precision (show statistical significance) and can be generalized to other similar populations (externally valid).
